# LH-induced Transcriptional Regulation of *Klf4* Expression in Granulosa Cells Occurs via the cAMP/PKA Pathway and Requires a Putative Sp1 Binding Site

**DOI:** 10.3390/ijms21197385

**Published:** 2020-10-06

**Authors:** Hyeonhae Choi, Jaesook Roh

**Affiliations:** Laboratory of Reproductive Endocrinology, Dept. of Anatomy and Cell Biology, College of Medicine, Hanyang University, Seoul 133-791, Korea; dalpu2002@hanyang.ac.kr

**Keywords:** *Klf4*, LH surge, PKA, *Sp1*, granulosa cells, luteinization, ovary

## Abstract

*Krüppel-like factor 4* (*Klf4*) plays an important role in the transition from proliferation to differentiation in a wide variety of cells. Previous studies demonstrated its critical role in the luteal transition of preovulatory granulosa cells (GCs). This study used cultured rat preovulatory GCs to investigate the mechanism by which luteinizing hormone (LH) regulates *Klf4* gene expression. *Klf4* mRNA and protein were rapidly and transiently induced by LH treatment, reaching peak levels after 45 min and declining to basal levels by 3 h. Pretreatment with the protein synthesis inhibitor cycloheximide had no effect on LH-stimulated *Klf4* expression, indicating that *Klf4* is an immediate early gene in response to LH. To investigate the signaling pathway involved in LH-induced *Klf4* regulation, the protein kinase A (PKA) and protein kinase C (PKC) pathways were evaluated. A-kinase agonists, but not a C-kinase agonist, mimicked LH in inducing *Klf4* transcription. In addition, specific inhibitors of A-kinase abolished the stimulatory effect of LH on *Klf4* expression. Truncation of a *Klf4* expression construct to −715 bp (p*Klf4*-715/luc) had no effect on transcriptional activity, whereas deletion to −402 bp (p*Klf4*-402/luc) dramatically reduced it. ChIP analysis revealed in vivo binding of endogenous Sp1 to the −715/−500 bp region and maximal transcriptional responsiveness to LH required the Sp1 binding element at −698/−688 bp, which is highly conserved in mice, rats, and humans. These findings demonstrate that *Klf4* is activated by LH via the cAMP/PKA pathway and a putative Sp1 binding element at −698/−688 bp is indispensable for activation and suggest that *Klf4* could be a target for strategies for treating luteal phase insufficiency induced by an aberrant response to the LH surge.

## 1. Introduction

The preovulatory luteinizing hormone (LH) surge induces the terminal differentiation of granulosa cells (GCs) and the follicular/luteal shift in steroidogenesis from estradiol to progesterone [1,2]. These changes require specific transcription factors that coordinate expression of diverse genes in preovulatory follicles [1,2]. Recently, we identified *Klf4* as a key regulator induced by the LH surge in the ovary [3]. *Klf4* is a member of the *Sp/Klf* transcription factor family and encodes a 55 kDa protein [4] that is 90 to 91% identical in humans, mice and rats.

*Klf4* is involved in fundamental cellular responses such as cell growth, proliferation, and differentiation [5,6,7]. It plays a role in the terminal differentiation of diverse non-gonadal cells [5,7,8] as well as Sertoli cells [9], and its mRNA is markedly increased in Graafian follicles relative to preantral follicles [10]. Similarly, expression of *Klf4* was rapidly and transiently induced in preovulatory GCs [3], supporting the functional relevance of the stage-specific expression of *Klf4* in cellular differentiation in the ovary. Recently, we also demonstrated that *Klf4* has a role in regulating the cell cycle of preovulatory GCs [11] as well as in modulating ovary-specific expression of aromatase [3]. Thus, increases in *Klf4* mRNA and protein in preovulatory GCs are responsible for the terminal differentiation of GCs by regulating the cell cycle as well as the luteal shift in steroidogenesis.

Clinical investigations point to downregulation of *Klf4* expression in the ovaries of patients with polycystic ovary syndrome (PCOS) [12]. In view of the involvement of *Klf4* in aromatase expression, downregulation of *Klf4* may be related to the inappropriate steroid synthesis found in PCOS patients. Therefore, *Klf4* may be a potential target for the development of therapeutic strategies in estrogen-related gynecological disorders as well as in luteal phase deficiency.

*Klf4* expression in diverse non-gonadal cells is enhanced by stimuli such as endothelin, IBMX, and estrogen [13,14,15]. On the other hand, its expression in gonads is dependent on gonadotropins. For instance, in primary mice Sertoli cell, FSH upregulates *Klf4* expression [16], and LH increases the abundance of *Klf4* transcripts in porcine granulosa–luteal cells [17]. Previously, we characterized the expression of both mRNA and protein for *Klf4* during the periovulatory period using ovaries from PMSG/hCG-primed immature mice and rats, and provided evidence for LH-initiated expression of *Klf4* in the GCs of preovulatory follicles [3].

Taken together, our observations indicate that the rapid and transient increases in *Klf4* expression in GCs are temporally associated with molecular events that define the termination of GCs differentiation and establishment of the luteal cell phenotype. Hence, the transcriptional machinery necessary for transactivation of *Klf4* must be present to respond to the LH surge. However, the cellular/molecular mechanisms by which LH modulates *Klf4* gene expression in the ovary are currently unknown. The present study aimed to explore these mechanisms. We first characterized the induction kinetics of *Klf4* mRNA and protein expression in cultured preovulatory rat GCs after LH treatment, and using this in vitro experimental model, we investigated the signal transduction pathway by which the LH surge induces *Klf4* expression.

## 2. Results

### 2.1. LH Induces Transient Klf4 Expression in Cultured Preovulatory GCs

**Figure 1:**
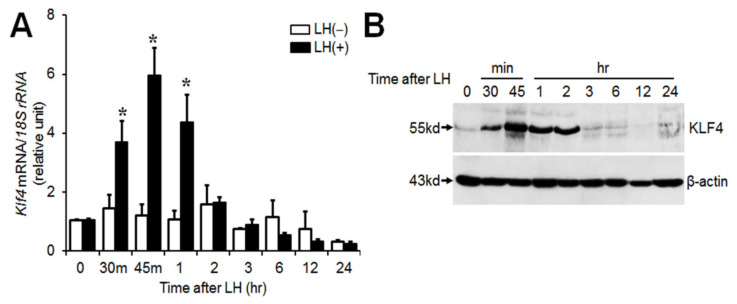
Regulation of *Klf4* expression by luteinizing hormone (LH) in cultured preovulatory granulosa cells (GCs). (**A**) Real-time RT-PCR analysis of *Klf4* mRNA levels. GCs obtained from rat preovulatory follicles (ovary at 48 h of PMSG) were cultured with or without LH (200 ng/mL) for the indicated times. *18S rRNA* was used to normalize reactions. Values were calculated as fold changes relative to the value at 0 h and are expressed as means ± SDs of at least three independent preparations of GCs. * *p* < 0.05 vs. 0 h; (**B**) Western blot analysis of KLF4 protein levels. Lysates were immunoblotted with anti-KLF4 antibody (1:1000) (abx006830, Abbexa Ltd.) and a representative blot from three independent experiments is shown. β-actin served as a loading control and was used for normalization. Arrows indicate bands corresponding to KLF4 (55 kDa) and β-actin (43 kDa), respectively.

We have previously demonstrated transient induction of *Klf4* in preovulatory follicles after the LH surge in the rat ovary [3]. In this study, we explored whether the transient increase in *Klf4* induced by LH/hCG in vivo could be reproduced in vitro. A large body of studies in animal models and tissue culture systems has established that preovulatory GCs luteinize in culture in response to addition of appropriate gonadotropins [18]. In addition, there was no difference in luteinization of GCs incubated with or without oocytes [19]. Hence, preovulatory GC culture systems might be a good model for such studies. Preovulatory GCs were treated with a luteinizing dose of LH (200 ng/mL) for the indicated times and were subjected to real time PCR and Western blot analysis. LH treatment induced a dramatic, transient increase in *Klf4* mRNA (Figure 1A). The mean fold changes at 30 and 45 min were 3.7- and 6-fold compared with untreated control (*p* < 0.01). In addition, Western blot analyses revealed maximum expression of KLF4 protein at 45 min, remaining elevated until 2 h following stimulation with LH (Figure 1B).

### 2.2. LH Modulates Klf4 Transcription via A cAMP/PKA-Dependent Signaling Pathway

**Figure 2:**
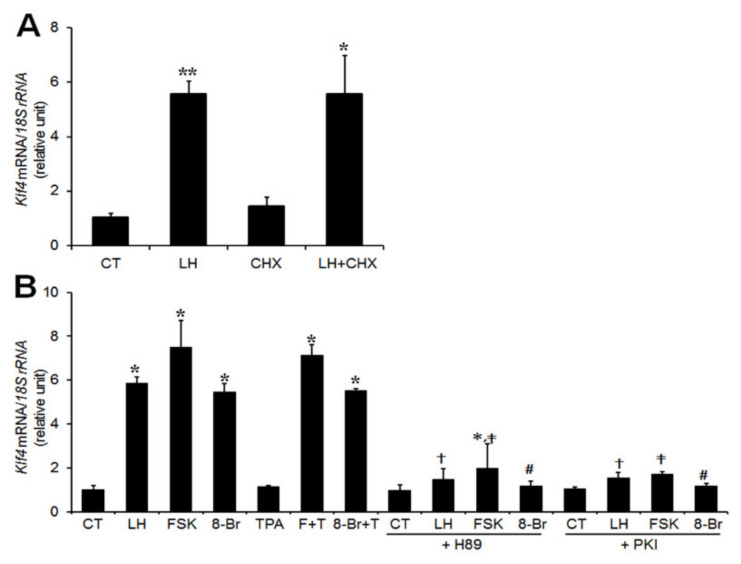
Regulation of *Klf4* gene expression by LH is mediated through the cAMP/PKA-dependent pathway in preovulatory GCs. (**A**) Real-time RT-PCR analysis of *Klf4* mRNA levels in GCs treated with either LH or CHX alone or both. GCs isolated from preovulatory follicles (48 h after PMSG) were incubated for 45 min in medium containing LH (200 ng/mL) with or without 30-min pretreatment with cycloheximide (CHX, 6 μg/mL). Relative levels of *Klf4* mRNA were normalized to the *18S rRNA*. Values were calculated as fold changes relative to untreated controls (CT) and are expressed as means ± SDs of at least three independent preparations of GCs. * *p* < 0.05, ** *p* < 0.01 vs. CT. (**B**) Real-time RT-PCR analysis of *Klf4* mRNA levels in GCs treated with PKA or PKC agonists and antagonists. Preovulatory GCs were preincubated with 0.1% DMSO (Control, CT), H89 (10 μM), or PKA inhibitor 14–22 Amide (PKI, 20 μM) for 30 min, and stimulated with LH (200 ng/mL), forskolin (FSK, 10 μM), 8-bromo-cyclic AMP (8-Br, 1 mM), 12-O-tetradecanoylphorbol-13-acetate (TPA, 30 nM), or FSK plus TPA or 8-Br plus TPA for 45 min. Relative levels of *Klf4* mRNA were normalized to the *18S rRNA*. Values were calculated as fold changes relative to the control and are expressed as means ± SDs of at least three independent preparations of GCs. FSK and 8-Br, PKA agonists; TPA, PKC agonist; H89 and PKI, specific inhibitors of PKA; F+T, FSK+TPA; 8-Br+T, 8-Br+TPA. * *p* < 0.05, ^∗∗^ *p* < 0.01 vs. CT; ^†^ *p* < 0.05, LH vs. LH+H89 or PKI; ^‡^ *p* < 0.05, FSK vs. FSK+H89 or PKI; ^#^ *p* < 0.05, 8-Br vs. 8-Br+H89 or PKI.

To determine whether *Klf4* was upregulated as an immediate early gene, we examined the effect of cycloheximide (CHX), an inhibitor of protein synthesis, on the induction of *Klf4* mRNA. GCs were preincubated with 6 μg/mL CHX for 30 min and then treated with LH (200 ng/mL) for 45 min. CHX treatment did not inhibit the LH-induced upregulation of *Klf4* mRNA levels, indicating that *Klf4* induction does not require de novo protein synthesis (Figure 2A).

**Figure 3:**
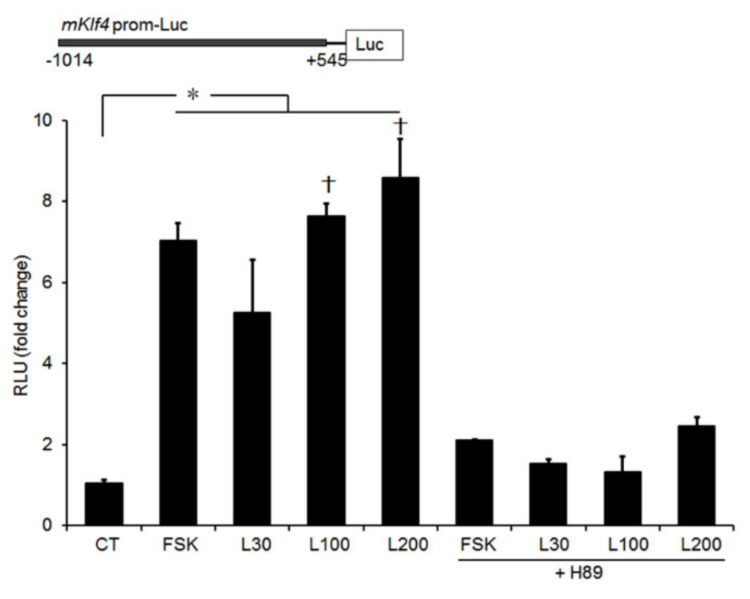
Transcriptional activity of the *Klf4* promoter induced by LH is inhibited by PKA inhibition. Preovulatory GCs were transiently transfected with a luciferase reporter construct containing region −1014/+545 bp of the mouse *Klf4* gene promoter (1 μg) (upper panel). At 24 h after transfection, cells were preincubated with DMSO or H89 (10 μM) for 30 min, and then treated with LH (30, 100, and 200 ng/mL) or FSK (20 μM) for 3 h. Cell lysates were assayed for luciferase, expressed as relative light units (RLU), and normalized to Renilla luciferase activity in transfected cells. Values are fold-changes relative to untreated control (CT), and are means ± SDs of four independent experiments, each performed in triplicate. L30, LH 30 ng/mL; L100, LH 100 ng/mL; L200, LH 200 ng/mL. * *p* < 0.05 vs. CT; ^†^ *p* < 0.05, vs. L30.

Since the LH surge activates both protein kinase A (PKA) and PKC signaling pathways in PO GCs [20], we examined the possible involvement of these signaling mediators in LH-induced *Klf4* upregulation. Preovulatory GCs were cultured with either the PKA agonist (forskolin or 8-bromo-cyclic-AMP), or the PKC agonist (TPA). The stimulatory effect of LH on *Klf4* mRNA was mimicked by both PKA agonists but not by the PKC agonist (Figure 2B), and combined treatment had no additive effect. To confirm that *Klf4* expression is induced by activation of the PKA pathway, GCs were treated simultaneously with agonist and potent PKA inhibitors. Addition of either H89 or PKA inhibitor 14–22 Amide (PKI) markedly reduced LH- and PKA agonists-induced *Klf4* mRNA levels (Figure 2B). Similar results were obtained using a luciferase reporter construct under the control of the mouse *Klf4* gene promoter (Figure 3).

### 2.3. LH-Induced Increases in Klf4 Promoter Activity Require Regulatory Elements between −715 and −402 bp of The 5′ region of The Klf4 Promoter

**Figure 4:**
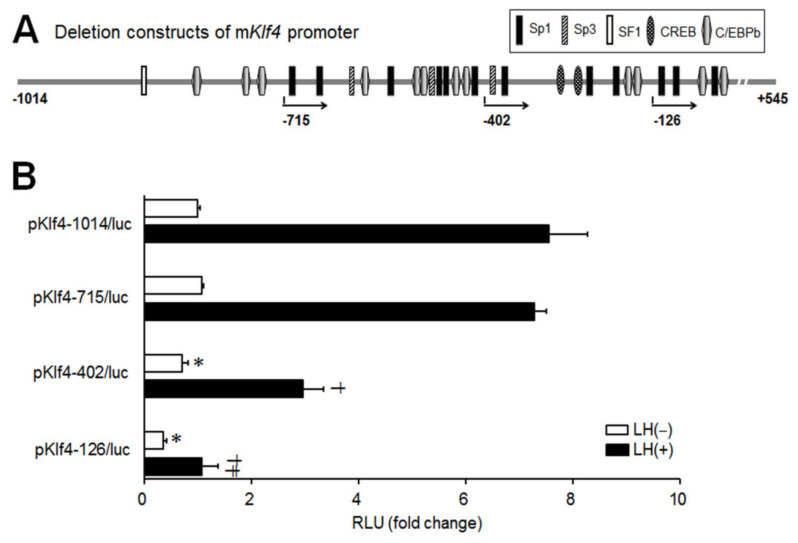
Sequential 5′-deletion analysis of the *Klf4* promoter. (**A**) Schematic representing the putative functional elements in the mouse *Klf4* promoter within the region −1014/+545 (base pairs are enumerated from the transcriptional start site). Sp1, specificity protein 1; Sp3, specificity protein 3; SF1, steroidogenic factor 1; CREB, cAMP responsive element binding protein; C/EBPβ, CCAAT enhancer binding protein β. The length and region of the deletion constructs that it spans are represented by the arrows. (**B**) Preovulatory GCs were transfected with pGL2-basic vectors containing −1014/, −715/, −402/, or −126/+545 bp of the mouse *Klf4* 5′-flanking region (designated as p*Klf4*-1014/luc, p*Klf4*-715/luc, p*Klf4*-402/luc, and p*Klf4*-126/luc, respectively) (1 μg). At 24 h after transfection, cells were treated with or without LH (200 ng/mL) for 3 h. Cell lysates were assayed for luciferase, expressed as relative light units (RLU), and normalized to Renilla luciferase activity. Cells transfected with the p*Klf4*-1014/luc construct, without any treatment, constituted the control group (CT). Values are fold changes relative to the control, and are means ± SDs of four independent experiments, each performed in triplicate. * *p* < 0.05 vs. CT; ^†^ *p* < 0.05, ^‡^ *p* < 0.01 vs. LH treated p*Klf4*-1014/luc.

To investigate the region of the *Klf4* promoter required for the transcriptional upregulation of *Klf4*, sequential 5′-deletion constructs of mouse *Klf4* promoter were generated as depicted in Figure 4A. The resulting constructs were transiently transfected into preovulatory GCs and tested for LH responsiveness (Figure 4B). For comparing luciferase activities, cells transfected with the p*Klf4*-1014/luc, without any treatment, constituted the control group. p*Klf4*-1014/luc and p*Klf4*-715/luc yielded 7.6- and 7.3-fold increases in luciferase activity by LH, respectively. Induction was significantly lower with the p*Klf4*-402/luc construct and deletion to −126 (p*Klf4*-126/luc) resulted in unresponsiveness to LH as well as a 60% decrease in basal activity. Evidently, the sequence between −715 to −402 bp is important for maximal induction of *Klf4* by LH, while the sequence between −402 to −126 bp is critical for basal transcription.

### 2.4. LH-Induced Upregulation of Klf4 Is Mediated by The Sp1 Binding Motif in GCs

**Figure 5:**
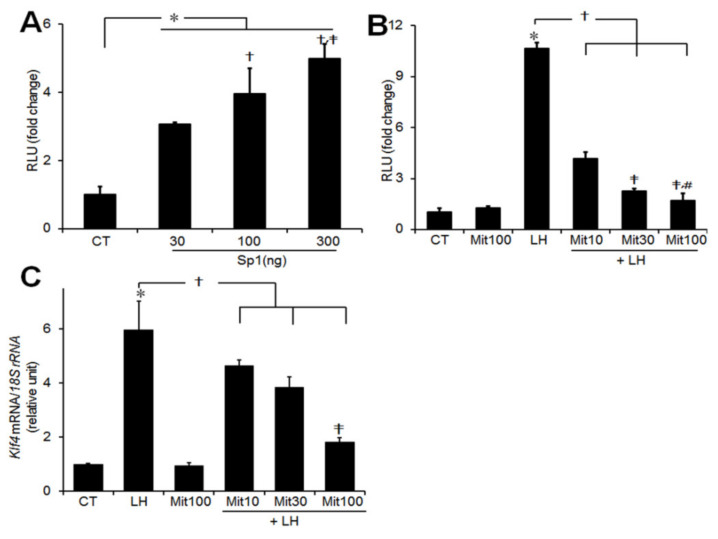
Transcriptional activity of the *Klf4* promoter induced by LH requires the Sp1 binding elements. (**A**) Preovulatory GCs were obtained from preovulatory follicles of rat ovaries 48 h after PMSG injection. GCs were cotransfected with 1 μg p*Klf4*-1014/luc reporter construct along with increasing amount of an *Sp1* expression plasmid (30, 100, and 300 ng) or empty vector. At 24 h after transfection, cells were treated with or without LH (200 ng/mL) for 3 h. Cell lysates were assayed for luciferase. Cells transfected with promoter only, without any treatment, constituted the control group (CT). Values are fold changes relative to the control, and are means ± SDs of three independent experiments, each performed in triplicate. * *p* < 0.05 vs. CT; ^†^ *p* < 0.05 vs. *Sp1* 30 ng; ^‡^ *p* < 0.01 vs. *Sp1* 100 ng. (**B**) Preovulatory GCs were transiently transfected with 1 μg p*Klf4*-1014/luc construct for 24–36 h. Where indicated, they were pretreated with increasing amounts of mithramycin (10, 30, and 100 nM) (Sp1 binding inhibitor) for 30 min, and then treated with LH (200 ng/mL) for 3 h. Values are fold changes relative to the control, and are means ± SDs of four independent experiments, each performed in triplicate. * *p* < 0.05 vs. CT; ^†^ *p* < 0.05 vs. LH; ^‡^ *p* < 0.05 vs. Mit10; ^#^ *p* < 0.05 vs. Mit30. (**C**) Preovulatory GCs were pretreated with increasing amounts of mithramycin (10, 30, and 100 nM) for 30 min, and then treated with LH (200 ng/mL) for 45 min. Relative levels of *Klf4* mRNA were normalized to the *18S rRNA*. Values were calculated as fold changes relative to the CT and are expressed as means ± SDs of at least three independent preparations of GCs. Mit10, mithramycin 10 nM; Mit30, 30 nM; Mit100, 100 nM. * *p* < 0.01 vs. CT; ^†^ *p* < 0.05 vs. LH; ^‡^ *p* < 0.05 vs. LH+Mit10.

The transcription factor *specificity protein-1* (*Sp1)* has been shown to play a role in the regulation of *Klf4* expression in non-ovarian cells [19,20]. To see whether LH-induced upregulation of *Klf4* is mediated by *Sp1* and its binding elements, we transiently cotransfected GCs with the p*Klf4*-1014/luc construct and increasing amounts of an *Sp1* expression plasmid. Overexpression of *Sp1* stimulated *Klf4* promoter activity, reaching approximately fivefold at 300 ng of *Sp1* (*p* < 0.05 vs. CT) (Figure 5A). To examine the role of the Sp1 binding motif in LH-induced transcription of *Klf4*, GCs transfected with a *Klf4* promoter construct were treated with mithramycin, an Sp1 binding inhibitor. As shown in Figure 5B, LH-induced luciferase activity was strongly inhibited by mithramycin, as was expression of *Klf4* mRNA (Figure 5C).

### 2.5. Sp1 Binds to The Klf4 Promoter Region in Vivo

**Figure 6:**
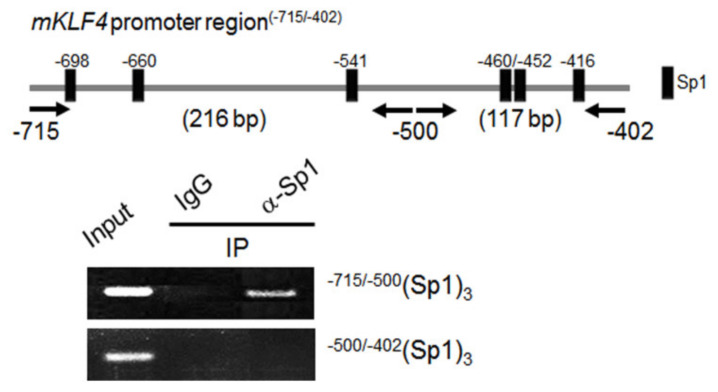
ChIP analysis of *Sp1* binding to the *Klf4* promoter in vivo. Preovulatory GCs were obtained at 1 h of hCG injection in PMSG-primed mice and chromatin was harvested for ChIP assays. One tenth of the chromatin was kept as input DNA control (Input) before immunoprecipitation. Immunoprecipitations (IP) were performed with Sp1 antibody (5 μg/mL) (sc-420, Santa Cruz Biotech.), or normal mouse IgG (1 μg/mL) (sc-2025, Santa Cruz Biotech.) as a negative control. DNAs were analyzed by PCR using the two sets of primer pairs indicated with arrows, i.e., one from −715 to −500 (216 bp) and the other from −500 to −402 (117 bp), each spanning three *Sp1* binding sites. The PCR products were electrophoresed in an agarose gel and visualized. A representative gel from three independent experiments is shown. Input, input DNA control; IgG, negative control.

To investigate potential interactions between Sp1 and the *Klf4* promoter, direct binding of Sp1 to the putative Sp1 binding sites in the endogenous *Klf4* promoter in vivo was analyzed by ChIP assays. We first focused on the sequence between −715 to −402 bp (relative to the transcription start site) in the *Klf4* promoter, and using a web-based transcription factor prediction program with a threshold cutoff of 0.90, identified six putative Sp1 binding sites (as depicted in Figure 6, upper lane). ChIP assays were performed using preovulatory GCs isolated from PMSG-treated immature mouse ovaries (1 h after injecting hCG), a time when *Klf4* was highly expressed (Figure S1). We determined whether endogenous Sp1 bound to the candidate elements in the *Klf4* promoter using primers that flanked nucleotides −715/−500 bp and −500/−402 bp from the transcription start site (Figure 6, upper lane). The PCR fragment (216 bp (−715/−500 bp)) containing the Sp1 binding sequences (−698, −660 and −541) was enriched in chromatin samples treated with Sp1 antibody, but no PCR fragment was amplified in the proximal region (−500/−402 bp) (Figure 6, lower panel). These results demonstrate in vivo binding of endogenous Sp1 to the *Klf4* promoter between positions −715 and −500.

### 2.6. Mutation of The Sp-1 Binding Site at −698/−688 Results in Loss of LH-Induced Klf4 Promoter Activity

**Figure 7:**
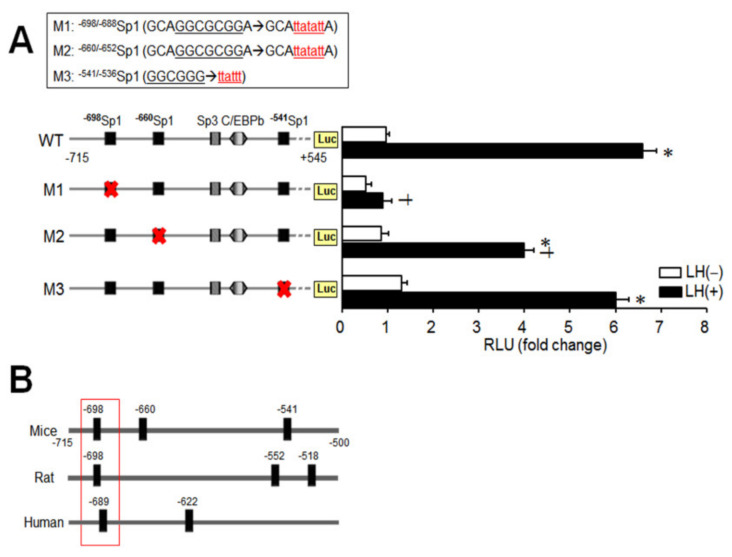
Loss of LH-induced *Klf4* promoter activity by mutation of the ^−698/−688^Sp1 binding site. (**A**) Site-directed mutants were prepared from p*Klf4*-715/luc and were designated M1 (p*Klf4*Δ^−698/688^*Sp1*/luc), M2 (p*Klf4*Δ^−660/652^*Sp1*/luc), and M3 (p*Klf4*Δ^−541/536^*Sp1*/luc) as shown on the upper panel with the mutated region underlined. Preovulatory GCs were transiently transfected with either the wild-type *Klf4* luciferase reporter (WT) or mutant versions in which one of the *Sp1* elements was mutated (M1, M2, or M3) (1 μg/well). At 24 h after transfection, cells were treated with (closed bar) or without LH (200 ng/mL) (open bar) for 3 h. Cell lysates were assayed for luciferase, expressed as relative light units (RLU), and normalized to Renilla luciferase activity in transfected cells. Cells transfected with wild-type construct, without any treatment, constituted the control group (CT). Values are fold-changes relative to the control, and are means ± SDs of four independent experiments, each performed in triplicate. * *p* < 0.05 vs. CT; ^†^ *p* < 0.05 vs. CT+LH. (**B**) The *Sp1* binding sites in the −715/−500 bp region of the *Klf4* promoter is aligned to homologous regions of the mice, rats, and humans. The conserved *Sp1* site is boxed with numbers indicating the relative position of the beginning of the *Sp1* site.

Since endogenous Sp1 binding was identified in the −715/−500 bp region of the *Klf4* promoter (Figure 6), the functional importance of the three Sp1 binding sites located within that region was analyzed. Site-directed mutants of p*Klf4*-715/luc were constructed and designated M1 (p*Klf4*Δ*^−^*^698/688^*Sp1*/luc), M2 (p*Klf4*Δ*^−^*^660/652^*Sp1*/luc), and M3 (p*Klf4*Δ*^−^*^541/536^*Sp1*/luc) (Figure 7A, upper panel). As summarized in Figure 7A, the Δ−698/688 bp deletion alone completely abolished LH-induced *Klf4* promoter activity and also led to a 50% decrease in basal promoter activity (*p*< 0.05). Disruption of the two other Sp1 sites (in M2 or M3) did not affect basal activity, but induced promoter activity was attenuated by 40% by deletion of the Sp1 site at −660/−652 bp (M2). These results indicate that the Sp1 binding element at −698/688 bp is required for both basal and LH-induced *Klf4* promoter activity in preovulatory GCs. Moreover, sequencing of the promoter revealed that this Sp1 binding motif is highly conserved in humans (NC_000009.12), mice (NC_000070.6), and rats (NC_005104.4) (Figure 7B).

## 3. Discussion

We showed previously that *Klf4*, a LH surge-induced factor, plays an important role in the coordinated transition from proliferation to differentiation of preovulatory GCs in response to the LH surge [3]. Here, we reported that the cAMP/PKA signaling pathway mediates LH-induced *Klf4* expression, and a Sp1 binding site at −689/−688 bp of the *Klf4* promoter, which is highly conserved in mice, rats, and humans, confers the LH responsiveness.

*Klf4* belongs to a small group of genes responding rapidly (within 15~60 min) to activation of signal transduction cascades [21]. Consistent with this, an ovulatory dose of LH induced rapid and transient expression of *Klf4* in cultured preovulatory GCs in vitro (Figure 1), as seen in vivo (Figure S1) [3]. Similarly, forskolin induced *Klf4* mRNA expression after 30 min in TM4 Sertoli cells, which peaked at 90 min [22]. On the other hand, it has been reported that peak induction of *Klf4* mRNA by LH occurred at 2 h and returned to basal after 6 h in porcine luteinized GCs [17]. These differences suggest that the response of *Klf4* expression to LH could differ depending on the stage of cell differentiation in the ovary.

KLF4 is rapidly degraded by the ubiquitin–proteasome complex [23] and contains a PEST domain [7], which is characteristic of proteins with half-lives of < 2 h [24]. Likewise, KLF4 protein levels declined very quickly after peaking at 45 min (Figure 1B), indicating that *Klf4* induction in GCs is an early response.

Although it has been shown that LH stimulates *Klf4* mRNA expression in porcine luteinized GCs [17], the molecular mechanism of its regulation in the ovary has not been studied. PKA, PI3K, PKC, and MEK5 have all been suggested as governing *Klf4* regulation in non-gonadal cells [7,13,14,15,23,25]. LH surge-induced differentiation and the expression of numerous genes in preovulatory follicles of GCs appeared to involve both the PKA and PKC pathways [20]. Therefore, we assessed whether either pathway is involved in LH-induced *Klf4* regulation using direct activators of the A-kinase (forskolin or 8-Bromo-cyclic AMP) or C-kinase (TPA). We showed that activation of PKA, but not PKC, mimicked the LH surge-induced increase in *Klf4* mRNA and promoter activity in preovulatory GCs (Figure 2 and Figure 4). Our data are consistent with studies showing that the cAMP/PKA pathway is responsible for upregulating *Klf4* transcription in Sertoli cells [22]. We also examined the PI3K/Akt signaling pathway and found that it was not involved in the LH surge-induced *Klf4* expression (Figure S2). Additionally, LH-induced *Klf4* induction was not affected by CHX (Figure 2), suggesting that it does not require de novo protein synthesis, as in other non-gonadal cells [15].

We also defined the main promoter element responsible for LH responsiveness, located in the −715/−402 bp region of the *Klf4* promoter (Figure 4B). Sequencing of this region revealed transcription factor binding sequences for Sp1, Sp3 and CAT/enhancer-binding protein-beta (C/EBPb) (Figure 4A). Although CREB binds and activates the *Klf4* promoter immediately after activation of the cAMP/PKA signal transduction pathway in other tissues [22], its binding site was not present in the −715/−402 bp region of the *Klf4* promoter (Figure 4A). Of note, six putative Sp1 binding motifs were found in that region.

*Sp1* is an ubiquitous transcription factor known to be involved in regulating a wide variety of genes [26] and constitutively expressed in ovarian GCs [27,28]. The expression of *Sp1* was rapidly stimulated when PKA was activated by LH treatment in rat preovulatory GCs (Figure S3), suggesting that *Sp1* may be one of the transcription factors induced by the LH surge. However, because increased *Klf4* expression occurred very rapidly following LH treatment of GCs and did not require new protein synthesis, the DNA binding and trans-activating activities of *Sp1* may be important for inducing *Klf4* expression. Since the activities of *Sp1* are modulated by cAMP/PKA signaling pathway in non-gonadal cells [29,30] that pathway might be involved in regulating its activity, and activated *Sp1* may bind to the *Klf4* promoter and activate it immediately after the LH surge.

Given the fact that many genes regulated in GCs by the LH surge have Sp1 binding sites and exhibit Sp1-dependent transactivation [27,31], the presence of multiple Sp1 binding sites in the −715/−402 bp region of the *Klf4* promoter suggested participation of *Sp1* in the upregulation of *Klf4* gene expression in GCs. Indeed, overexpression of *Sp1* transactivated *Klf4* promoter activity in a dose-dependent manner in preovulatory GCs (Figure 5A) as in non-gonadal cells [8,32,33].

In addition, important roles of Sp1 binding sites in *Klf4* transcriptional activities have been demonstrated in colon cancer cells and smooth muscle cells [25,32]. Consistent with this, inhibition of Sp1 binding reduced expression of *Klf4* in GCs (Figure 5B). We therefore propose that the Sp1 binding motif plays a critical role in integrating the transcriptional response of *Klf4* to the LH surge. ChIP analysis confirmed direct binding of Sp1 to the endogenous *Klf4* promoter containing the three Sp1 binding sites in the −715/−500 bp region in intact chromatin in response to LH treatment (Figure 6). On the other hand, the Sp1 binding sites located at −145 or −75 bp from the TSS have been reported to play a role in regulating *Klf4* expression in non-gonadal cell [25,33]. Thus the critical regions of the *Klf4* promoter for interaction with Sp1 may be different depending on the type of cells. On the other hand, *Sp3*, another member of the *Sp/Klf* family, is ubiquitously expressed in ovarian GCs and Sp3 recognizes the same sequences as Sp1 [34]. Hence, we also determined whether endogenous Sp3 bound to the candidate elements in the Klf4 promoter and found no interaction between them (Figure S4).

We also found that a conserved Sp1 binding site at −698/−688 bp in the *Klf4* promoter was indispensable for basal as well as LH-stimulated *Klf4* transcription in GCs (Figure 7A). In contrast, one/three consensus Sp1 binding sites between −150/+1 bp of the *Klf4* promoter have shown to be important for PDGF- or butyrate-induced *Klf4* expression in VSMCs [32], colon cancer cells [25], or odontoblast [33]. As stated above, the location of Sp1 binding domains required for the increased expression of *Klf4* in GCs was different from in non-gonadal cells, but Sp1 binding sites seem to be critical in transcriptional control of *Klf4* in diverse cells. However, other possible mechanism may affect the induction of *Klf4* by LH, and the involvement of other transcriptional regulators including other *Klf* family, which may interact with Sp1 binding site, remains to be determined.

## 4. Materials and Methods

### 4.1. Animals and Reagents

Twenty-one-day-old immature female Sprague Dawley rats were obtained from Samtako Biokorea (Kyunggi-do, South Korea) and housed under controlled conditions (22–24 °C, humidity 40–50%, 12 h light–dark cycle), with free access to food and water. The rats were allowed to acclimate until 26 days of age before being subjected to experimental conditions. Animal care was consistent with institutional guidelines, and the Hanyang University ACUC committee approved all animal procedures (HY-IACUC-16-0013) (24 Feb 2014). Ovine LH (NIH-LH-23) was obtained from the National Hormone and Pituitary Distribution Program (Baltimore, MD, USA). McCoy’s 5a medium (modified), Leibovitz L-15 medium, L-glutamine, penicillin-streptomycin, and fetal bovine serum (FBS) were from Life Technologies (Santa Clara, CA, USA). Unless otherwise indicated, reagents were purchased from Sigma (St. Louis, MO, USA).

### 4.2. Preparation and Culture of Preovulatory Granulosa Cells

Animals aged 26 days (body weight 55–60 g) were injected intraperitoneally with 10 IU PMSG to induce growth of multiple preovulatory follicles. Forty-eight hours later ovaries were dissected, and preovulatory follicles were punctured to obtain GCs. Ovarian debris and small follicles were removed by low-speed centrifugation at 500× *g* for 10 min and the remaining medium containing GCs was collected. The GCs were dispersed by repeated washing and suspended in culture medium (McCoy’s 5a supplemented with 2 mM L-glutamine, 100 U/mL penicillin, and 100 μg/mL streptomycin). Approximately 2 × 10^4^ viable cells were plated in 24-well culture dishes (Nunc 142475; Thermo Fisher Scientific, Waltham, MA, USA) and incubated at 37 °C in a humidified atmosphere with 5% CO_2_. After 2 h, the medium was replaced with fresh, serum-free medium, and hormones, agonists, and inhibitors were added as indicated in figure legends. At the end of experiments, GCs were frozen for total RNA or protein extraction.

### 4.3. Plasmids

The *specificity protein-1* (*Sp1*)-encoding plasmid was a gift from Prof. Guntram Suske (Addgene plasmid # 24543; http://n2t.net/addgene:24543; RRID:Addgene_24543) and the pGL2-1014 *Klf4*-promoter-luciferase construct was kindly provided by Prof. Vincent W. Yang (Emory University School of Medicine, Atlas, GA). Truncated *Klf4* promoter constructs (p*Klf4*-715/luc, p*Klf4*-402/luc, and pKlf4-126/luc) were generated by PCR-based subcloning. *Klf4* promoter constructs with mutated *Sp1* (at −698/−688, −660/−652, or −541/−536 bp) sequences were generated from p*Klf4*-715/luc using a QuickChange site-directed mutagenesis kit (Stratagene, La Jolla, CA). Primers were designed with the Stratagene web-based QuickChange primer design program as follows; for the *Sp1* site at −698/−688 bp, 5′-ACGCGTAAGAGCTCGGTACCCGAGCCCCAAAGTCAACGAA-3′ (sense) and 5′-TTCGTTGACTTTGGGGCTCGGGTACCGAGCTCTTACGCGT-3′ (antisense), for the *Sp1* site at −660/−652 bp, 5′-AACATAACCCGGGAGGTACCGGCCGCTCTCTTTCATAGCA-3′ (sense) and 5′-TGCTATGAAAGAGAGCGGCCGGTACCTCCCGGGTTATGTT-3′ (antisense), and for the *Sp1*site at −541/−536 bp, 5′- ACGCGTAAGAGCTCGGTACCCGAGCCCCAAAGTCAACG -3′ (sense) and 5′-CGTTGACTTTGGGGCTCGGGTACCGAGCTCTTACGCGT-3′ (antisense). The mutant constructs are shown in Figure 7A (upper panel): (p*Klf4*Δ^−698/688^*Sp1*/luc, M1) 5′-GCAGGCGCGGA-3′→5′-GCAttatattA-3′, (p*Klf4*Δ^−660/652^*Sp1*/luc, M2) 5′-GCAGGCGCGGA-3′→5′-GCAttatattA-3′, and (p*Klf4*Δ^−541/536^*Sp1*/luc, M3) 5′-GGCGGG-3′→5′-ttattt-3′. Methylated parental DNA templates were digested with DpnI at 37 °C for 1 h and purified. Mutations were confirmed by direct sequencing.

### 4.4. Transient Transfection and Luciferase Reporter Assays

For transient transfections, GCs were prepared as described above and resuspended in electroporation buffer (MPK1025; Thermo Fisher Scientific, Waltham, MA, USA). They were mixed with plasmids as indicated in the figure legends, and electroporated with a single pulse of 1000 V, 40 ms, using a Neon™ Transfection System (MPK5000, Thermo Fisher Scientific, Waltham, MA, USA). The choice of these conditions was based on the results of preliminary optimization experiments that achieved a transfection efficiency of approximately 70%. The transfected cells (1 × 10^5^ cells/well) were resuspended in culture medium and plated in 24-well culture plates. Six hours later, this medium was replaced with fresh culture medium and the cells were cultured for 24–36 h at 37 °C in a humidified atmosphere with 5% CO_2_ with/without further treatment. At the end of the experiment, the cells were collected for total RNA/protein extraction or luciferase assay. For luciferase assay, cells were washed with PBS, Reporter Lysis Buffer (100 μL) (Promega Corp., Madison, WI) was added to each well and 20 μL of each supernatant was used for luciferase determination using a luminometer (FB12, Berthold Technologies, Bad Wildbad, Germany). Firefly luciferase activities were normalized by Renilla luciferase activities and data are expressed as means ± standard deviations (SD) of triplicate measurements in four independent experiments.

### 4.5. Real Time Quantitative PCR

Total RNA was isolated with a RNeasy extraction kit (Qiagen Inc., Valencia, CA, USA). Samples (1–2 µg) of total RNA were annealed (5 min at 70 °C) to oligo(dT)_18_ primer and reverse transcribed using cDNA synthesis platinum master mix (GenDEPOT, Katy, TX). Primers were designed with the Primer-BLAST program (NCBI, Bethesda, MD) and were as follows: *Klf4* forward, 5′-GAGAGGAACTCTCTCACATGAAGC-3′ and reverse, 5′-AAGGATAAAGTCTAGGTCCAGGAGA-3′ (NM_053713.1); *Sp1* forward, 5′- ACAACTTTCACAGGGTGCCA-3′ and reverse, 5′- AGAGACTGTGCGGTTCTTGG-3′ (NM_012655.2). Amplified *18S ribosomal RNA* (*18S rRNA*) (forward, 5′-CGCGGTTCTATTTTGTTGGT-3′ and reverse, 5′-AGTCGGCATCGTTTATGGTC-3′) (M11188.1) was used to normalize each reaction (amplification product sizes 185, 136, and 218 bp for *Klf4*, *Sp1*, and *18S rRNA*, respectively). Real-time PCR reactions were carried out in total volumes of 20 μL, with Prime Q-Master Mix (with SYBR Green I) (GeNet Bio Inc., Daejeon, South Korea) using a LightCycler 480 II System (Roche Molecular Diagnostics, Indianapolis, IN, USA). The PCR cycle conditions were: 10 min at 95 °C, 45 cycles of 95 °C for 10 s, 58~64 °C for 10 s, and 72 °C for 10 s. Samples were run in triplicate (Roche) and mean values were compared with the control values to calculate relative amounts of transcript. Data are expressed as means ± SDs of duplicate or triplicate measurements in four independent experiments.

### 4.6. Western Blot Analysis

GCs were harvested 24–36 h after transfection and washed with cold PBS before lysis in Laemmli buffer containing β-mercaptoethanol. Lysates were boiled for 5 min. Samples of 30 μg were loaded and resolved by 10% SDS-PAGE gel electrophoresis, and proteins were transferred to nitrocellulose membranes (Amersham Pharmacia Biotech, Arlington Heights, IL). Membranes were blocked for 2 h at room temperature (RT) in TBS-0.1% Tween containing 5% fat-free dry milk, and incubated at 4 °C overnight with anti-KLF4 antibody (abx006830, Abbexa Ltd., Cambridge, UK) or anti-Sp1 antibody (sc-420, Santa Cruz Biotechnology Inc., Dallas, TX, USA) diluted 1:1000 and 1:200, respectively. The membranes were washed with TBS-0.1% Tween and then incubated with peroxidase-conjugated donkey anti-rabbit secondary antibody (1:8000) (Boehringer Mannheim, Indianapolis, IN, USA) for KLF4, and with peroxidase-conjugated goat anti-mouse secondary antibody (1:2500) (W402B, Promega, Madison, WI, USA) for *Sp1*. After 2 h, immunolabeled proteins were detected with an enhanced chemiluminescence kit (Amersham Pharmacia Biotech., Little Chalfont, UK). The 55 kDa KLF4 and 106 kDa Sp1 proteins are indicated in the figures. To ensure that lysates were loaded equally, the blots were stripped and incubated with β-actin (1:3000) (ab8227, Abcam, Cambridge, UK). Data were collected from three independent experiments.

### 4.7. Chromatin Immunoprecipitation (ChIP) Assays

ChIP assays were performed using a ChIP kit (Upstate Biotechnology, Inc., Lake Placid, NY) according to the manufacturer’s protocol with minor modifications. Briefly, preovulatory GCs (2 × 10^6^ cells) collected from the ovaries 1 h after injecting hCG into PMSG-primed mice were cross-linked with 1% formaldehyde for 10 min at room temperature. Reactions were terminated by adding glycine (final concentration, 0.125 M) for 5 min at room temperature. Cells were pelleted by centrifugation and lysed in 1 mL ice-cold lysis buffer containing protease inhibitor cocktail (Roche Applied Science, Indianapolis, IN, USA). The lysates were sonicated on ice at 250 W for 25 min with 15 sec sonications at 30 sec intervals with a Bioruptor KR (CosmoBio Co., Ltd., Tokyo, Japan) to obtain DNA fragments of an average length of approximately 100–500 bp. Chromatin was immunoprecipitated overnight at 4 °C with anti-Sp1 antibody (5 μg/reaction) (Santa Cruz Biotech) or normal mouse IgG (1 μg/reaction) (Santa Cruz) as a negative control. Immune complexes were collected with protein G-agarose slurry for 2 h at 4 °C with rotation and washed for 3 min each in low salt wash buffer, high salt wash buffer, LiCl wash buffer, and Tris/EDTA buffer (twice). Precipitates were then extracted twice with elution buffer. Eluates were pooled, and cross-linking was reversed by incubation at 65 °C overnight. Unbound proteins were digested with proteinase K (Promega Corp., Madison, WI) for 2 h at 45 °C, and chromatin was purified using a DNA Clean-up kit (GeneAll Biotech., Seoul, Korea). DNA was amplified using two set of primers spanning, respectively, the proximal three *Sp1* sites (the region between −500 and −402 bp) and the distal three *Sp1* sites (the region between −715 to −500 bp) in the *Klf4* promoter: the −500/−402 bp region (forward 5′-TGCGCAGACGACAGGAC-3′, reverse 5′-GCTCGAAAGTCCTGCCACG-3′) and the −715/−500 bp region (forward 5′-GGCCGCTCTCTTTCATAGCA-3′, reverse 5′-ACTCGAGAGCGCGATTATCC-3′) (Figure 6). After 25- to 28-cycles of amplification, the PCR products were run on a 1.5% agarose gel, stained with ethidium bromide, and visualized under UV light.

### 4.8. Statistical Analysis

All data were analyzed with IBM SPSS Statistics 21 for Windows (IBM Corp., Armonk, NY, USA). Statistical significance was determined by Kruskal–Wallis one-way analysis of variance for multiple group comparisons, and with the Mann–Whitney *U*-test for two-group comparisons. Data are expressed as means ± SDs of at least three independent experiments. Significance was accepted at *p* < 0.05.

## Supplementary Materials

Supplementary materials can be found at https://www.mdpi.com/1422-0067/21/19/7385/s1.
