# Supplementary material for: LH-induced Transcriptional Regulation of Klf4 Expression in Granulosa Cells Occurs via the cAMP/PKA Pathway and Requires a Putative Sp1 Binding Site
Source: Int J Mol Sci. 2020 Oct 6;21(19):7385. doi: 10.3390/ijms21197385 (PMC7582263; doi:10.3390/ijms21197385)
Supplement: Supplementary file 1 [file ijms-21-07385-s001.pdf]

# Supplementary figures

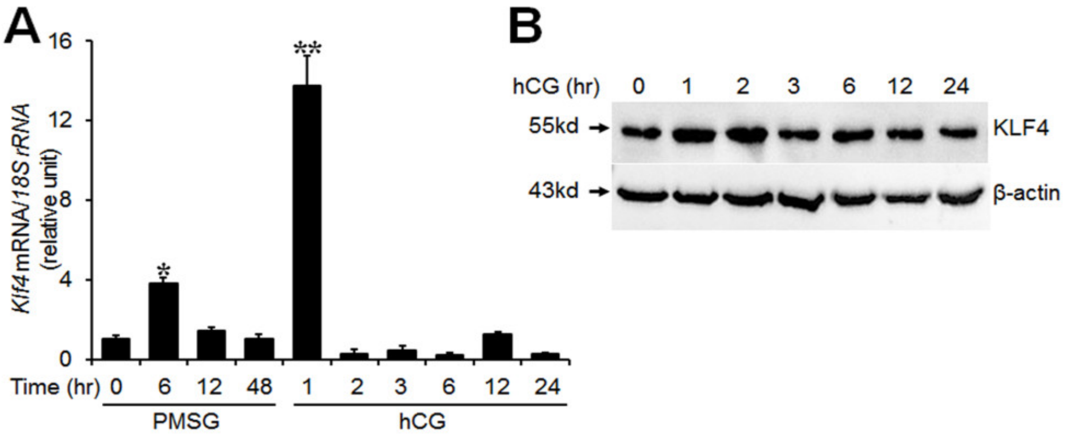


**Figure S1.** In vivo regulation of *Klf4* expression by gonadotropins in rat ovaries. *Klf4* mRNA **(A)** and protein levels **(B)** were analyzed by real-time RT-PCR and Western blot analyses. Periovulatory ovaries were obtained from PMSG-primed immature rats at the indicated times before and after hCG injection. *18S rRNA* was used to normalize the reactions. Bars indicate fold changes from the value at 0 h (before PMSG injection) and are means ± SDs of four to five animals per time point. *p< 0.05, **p< 0.01 vs. 0 h. Ovarian protein extracts were immunoblotted with anti-KLF4 antibody (abx006830, Abbexa Ltd.) and a representative blot is shown. β-actin served as a loading control and was used for normalization. Arrows indicate bands corresponding to KLF4 (55 kDa), and β-actin (43 kDa), respectively.

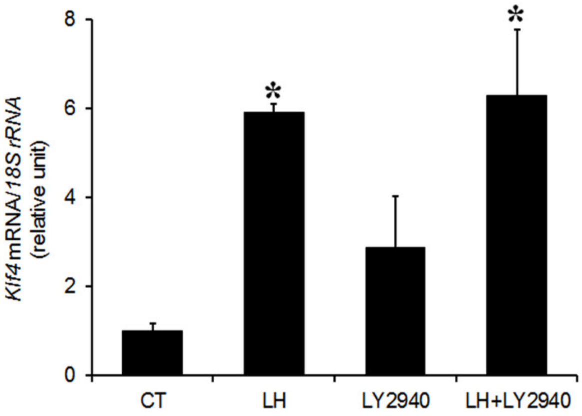


**Figure S2.** Effect of PI3K inhibitor in the regulation of LH-induced Klf4 expression in GCs. Real-time RT-PCR analysis of *Klf4* mRNA levels in GCs treated with PI3K inhibitor. Preovulatory GCs were preincubated with 0.1% DMSO (Control, CT) or LY2940 (25 μM) for 30 min, and stimulated with LH (200 ng/ml) for 45 min. Relative levels of *Klf4* mRNA were normalized to the *18S rRNA*. Values were calculated as fold changes relative to the control and are expressed as means ± SDs of three independent preparations of GCs. LY2940, specific inhibitor of PI3K. *p< 0.05 vs. CT.

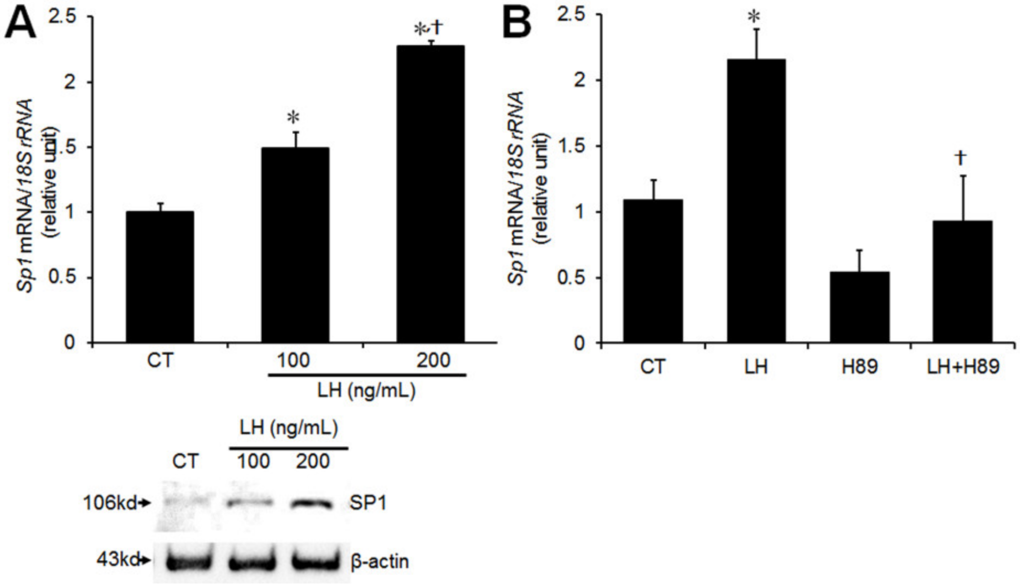


**Figure S3.** LH/PKA signaling participates in the regulation of *Sp1* expression in GCs. **(A)** *Sp1* mRNA is shown in the upper panel and protein expression in the lower panel. Preovulatory GCs were cultured with or without LH (0, 100, and 200 ng/ml) for 45 min and total RNA and protein were measured. To normalize reactions, *18S rRNA* and β-actin were used for real time RT-PCR and Western blot analysis, respectively. For Western blot, lysates were immunoblotted with anti-Sp1 antibody (1:200) (sc-420, Santa Cruz Biotech.) and a representative blot from three independent experiments is shown. Values were calculated as fold changes relative to the untreated control and are expressed as means ± SDs of at least three independent preparations of GCs. Arrowheads indicate bands corresponding to Sp1 (106 kDa) and β-actin (43 kDa). CT, untreated cells. *$p < 0.05$ vs. CT; †$p < 0.05$ vs. LH (100ng/ml). **(B)** Preovulatory GCs were preincubated with medium alone or H89 (10 μM) for 30 min, and then treated with LH (200 ng/ml) for 45 min. Relative levels of *Sp1* mRNA were normalized to the *18S rRNA*. Values were calculated as fold changes relative to untreated controls (CT) and are expressed as means ± SDs of at least three independent preparations of GCs. *$p < 0.05$ vs. CT; †$p < 0.05$ vs. LH.

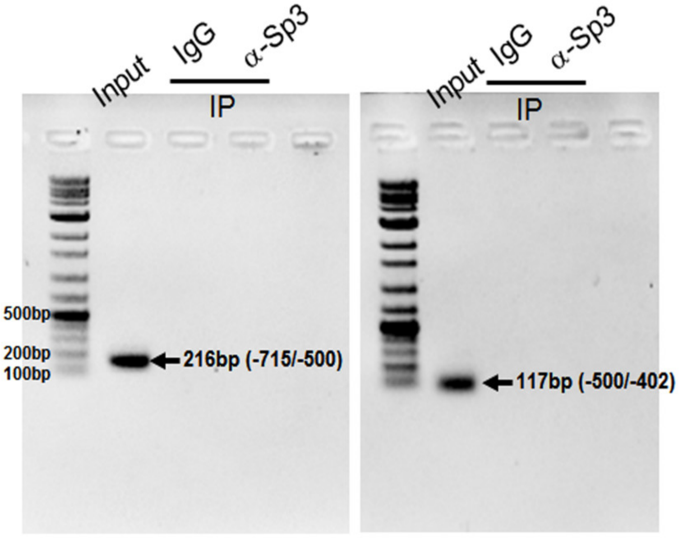


**Figure S4.** Gel picture from ChIP analysis of *Sp3* binding to the *Klf4* promoter *in vivo.* Preovulatory GCs were obtained at 1 h of hCG injection in PMSG-primed mice and chromatin was harvested for ChIP assays. One tenth of the chromatin was kept as input DNA control (Input) before immunoprecipitation. Immunoprecipitations (IP) were performed with Sp3 antibody (5 μg/ml)(sc-

28305, Santa Cruz Biotech.), or normal mouse IgG (1 μg/ml)(sc-2025, Santa Cruz Biotech.) as a negative control. DNAs were analyzed by PCR using the two sets of primer pairs indicated with arrows, i.e., one from -715 to -500 (216 bp) and the other from -500 to -402 (117 bp) each spanning three *Sp1*-binding sites. The PCR products were electrophoresed in an agarose gel and visualized. Input, input DNA control; IgG, negative control.
